# A systematic pan-cancer analysis of PXDN as a potential target for clinical diagnosis and treatment

**DOI:** 10.3389/fonc.2022.952849

**Published:** 2022-08-02

**Authors:** Xiaohu Zhou, Qiang Sun, Chang Xu, Zheng Zhou, Xiaoquan Chen, Xiuping Zhu, Zhaoshuai Huang, Weilin Wang, Yanjun Shi

**Affiliations:** ^1^ Department of Hepatobiliary and Pancreatic Surgery, Second Affiliated Hospital, Zhejiang University School of Medicine, Hangzhou, China; ^2^ Key Laboratory of Precision Diagnosis and Treatment for Hepatobiliary and Pancreatic Tumor of Zhejiang Province, Second Affiliated Hospital, Zhejiang University School of Medicine, Hangzhou, China; ^3^ Department of Head and Neck Surgery, Centre of Otolaryngology-Head and Neck Surgery, Zhejiang Provincial People’s Hospital, People’s Hospital of Hangzhou Medical College, Hangzhou, China; ^4^ Department of pediatric, Second Affiliated Hospital, Zhejiang University School of Medicine, Hangzhou, China; ^5^ Department of Pharmacy, Second Affiliated Hospital, Zhejiang University School of Medicine, Hangzhou, China

**Keywords:** PXDN, cancer, mutation, methylation, tumor microenvironment, immune infiltration

## Abstract

Peroxidasin (PXDN), also known as vascular peroxidase-1, is a newly discovered heme-containing peroxidase; it is involved in the formation of extracellular mesenchyme, and it catalyzes various substrate oxidation reactions in humans. However, the role and specific mechanism of PXDN in tumor are unclear, and no systematic pan-cancer studies on PXDN have been reported to date. This study employed data from multiple databases, including The Cancer Genome Atlas and The Genotype-Tissue Expression, to conduct a specific pan-cancer analysis of the effects of PXDN expression on cancer prognosis. Further, we evaluated the association of PXDN expression with DNA methylation status, tumor mutation burden, and microsatellite instability. Additionally, for the first time, the relationship of PXDN with the tumor microenvironment and infiltration of fibroblasts and different immune cells within different tumors was explored, and the possible molecular mechanism of the effect was also discussed. Our results provide a comprehensive understanding of the carcinogenicity of PXDN in different tumors and suggest that PXDN may be a potential target for tumor immunotherapy, providing a new candidate that could improve cancer clinical diagnosis and treatment.

## 1 Introduction

Recent epidemiological studies have reported an annual increase in the incidence of malignant tumors, and the latest research predicts that cancer will overtake ischemic heart disease as the leading cause of death by 2060 (1). In addition to the use of traditional surgical treatments, radiotherapy, and chemotherapy, immunotherapy has been the focus of extensive research in recent years (2). Numerous immunotherapy drugs are being applied clinically, and remarkable results are being observed. However, immunotherapy is associated with obvious complexities and uncertainties, and an active immune system can cause adverse reactions. Thus, highly effective immune checkpoints need to be discovered (3). The development of several tumor databases, such as TCGA and GTEx, has facilitated pan-cancer analyses of the molecular mechanisms of different tumors and their associated impacts on prognosis, aiding in the exploration of new tumor immunotherapy targets (4, 5).

Peroxidasin (PXDN), also known as vascular peroxidase-1 (VPO1), is a heme-containing peroxidase. PXDN is the only known enzyme capable of stabilizing basement membranes by catalyzing the formation of sulfilimine bonds between type IV collagen fibrils, which are only found within the basement membrane. Loss of PXDN function leads to a lack of type IV collagen fibril cross-linking and subsequent basement membrane instability, which suggests that PXDN plays a critical role in tissue development and homeostasis (4). In addition, early studies of PXDN suggest that mutations in this gene may lead to certain serious eye diseases (6).

Many recently conducted studies have found that PXDN plays an important role in tumors because it catalyzes the oxidation of numerous substrates through the intake of hydrogen peroxide (H2O2) to promote peroxidation (7). Some studies have also found that PXDN serves as a novel target for the redox sensitive transcription factor NRF2 (8), which plays an important role in tumorigenesis and development (9, 10). PXDN may also contribute to tumor proliferation, invasion, and migration in ovarian cancer by regulating the PI3K/Akt pathway (11). However, the role and specific mechanism of PXDN in tumors are unclear.

Therefore, we conducted a systematic pan-cancer analysis of PXDN using data from TCGA, GTEx, and other related databases to elucidate the effects of PXDN on tumor prognosis and related clinical characteristics. Additionally, we also investigated the association of PXDN with gene mutation and methylation, as well as the possible molecular mechanisms that may play a role in specific tumors. Furthermore, we discussed the effects of PXDN on the tumor microenvironment, including the infiltration of fibroblasts and immune cells. Our findings may provide new strategies for the treatment of clinically related tumors in the future.

## 2 Methods

### 2.1 Data acquisition and processing

Data were obtained from UCSC Xena (https://Xena.UCSC.edu/), which is an online database containing gene expression profiles and clinical and phenotypic data. We downloaded relevant gene expressions and clinical data of 33 tumors and corresponding normal samples from TCGA database, and gene expression data of 31 different normal tissues were obtained from the GTEx database (www.gtexportal.org/). All gene expression data were normalized by log2 transform. The differences between tumors and normal tissues were determined using t-tests. Kaplan-Meier curves, log-rank tests, and Cox proportional risk models were used to conduct all survival analyses. The correlations between variables were calculated using a Pearson or Spearman test. All statistical analyses were performed using R software (version 4.1.3, https://www.r-project.org); P < 0.05 was considered statistically significant.

### 2.2 Analysis of PXDN expression

Based on the transcriptome gene expression data of all tumor tissues and their corresponding normal tissues obtained from TCGA and GTEx, the differences in PXDN expression between the tumor and normal tissues in 31 tumor types were analyzed using the “Limma” package of R software. We then ranked the PXDN expressions of the 33 tumor types obtained from the TCGA database from low to high, and visualized them as box plots using the R packet “ggplot2”. The PXDN expression levels of normal tissues from the GTEx database were visualized using the same approach.

The UALCAN portal is an online tool that can be used to conduct proteome analyses of several cancer types. We used the Clinical Proteomics Tumor Analysis Consortium (CPTAC) data set to conduct a protein expression analysis, and the expression of PXDN in tumors and normal tissues was analyzed by entering “PXDN”. We selected protein expression datasets of the following 10 available tumors: breast cancer, ovarian cancer, colon cancer, clear cell renal cell carcinoma (RCC), Uterine Corpus Endometrial Carcinoma (UCEC), lung cancer, pancreatic cancer, head and neck carcinoma, glioblastoma, and liver cancer. To further verify PXDN expression differences at the protein level, we used the Human Protein Atlas (HPA; http://www.proteinatlas.org/) to download and compare differences in PXDN immunohistochemical staining results between six tumor types and normal tissues.

### 2.3 Prognostic survival analysis of PXDN

We combined the data of the expression level and the corresponding clinical traits of PXDN in each sample obtained from the TCGA database and used the following four parameters to study the effect of PXDN on prognosis: overall survival (OS), disease-specific survival (DSS), disease-free interval (DFI), and progression-free interval (PFI). A single factor Cox analysis was conducted using the R packages “survival” and “survminer,” and the results were visualized using the R package “forestplot.” The relevant forest map and related Kaplan-Meier survival curve were drawn based on the p values.

To study the effect of PXDN expression on tumor stages, we integrated data on the 33 tumor types as follows: Stage 1 was integrated with Stage 2 and Stage 3 integrated with that Stage 4. We used the R package “boxplot” to visualize the results.

### 2.4 PXDN methylation analysis

The cBioPortal website (https://www.cbioportal.org/) is an interactive exploration dataset containing data on multiple cancer genomics. We downloaded the data on PXDN methylation levels in 33 tumors from HM450 data and then integrated the PXDN expression data of each sample to explore the correlation between PXDN and methylation. We visualized the results using the R package “lollipop.” In addition, we continued to explore the impact of methylation on tumor prognosis using clinically relevant data and by selecting OS as a metric and plotting the associated Kaplan-Meier survival curves.

### 2.5 Analysis of PXDN mutation

We used the cBioPortal website to analyze PXDN mutations by selecting “TCGA pan-cancer Atlas study” in “Quick by Gene” and then typing “PXDN” for mutation-related properties. The associated gene mutation results were observed in the “Cancer Type Summary” module for all tumors in the TCGA data. Information about the PXDN mutation site was displayed using a protein structure diagram or as a three-dimensional (3D) structure using the “Mutation” module.

The correlations between PXDN and tumor single nucleotide variation (SNV) and copy number variation (CNV) were also explored. SNV has been found to be often closely associated with the tumor immune microenvironment, and it often serves as a prognostic marker for immune checkpoint inhibitors (12). Studies on CNV have found that it is positively correlated with patient susceptibility to tumors (13). The GSCA lite database (http://bioinfo.life.hust.edu.cn/GSCA/) was used to continue to explore PXDN-related mutations in tumors. In the “Mutation” module, all tumors were selected and PXDN was input, and the associated SNV and CNV statuses of this gene within tumors were obtained in turn.

### 2.6 GSEA and GSVA analysis

We used a Gene Set Enrichment Analysis (GSEA) to explore the possible enrichment pathway of PXDN in different tumors. Gene Ontology (GO) gene sets were downloaded from the GSEA website (https://www.GSEA-msigdb.org/GSEA/downloads.jsp), and the R package “ClusterProfiler” was used to analyze the PXDN function. A top-ranked enrichment pathway was obtained, where P < 0.05 was considered statistically significant.

We then analyzed the possible molecular mechanisms associated with PXDN in tumors. We used the HALLMAKER gene set from the MSigDB database, which included 50 enriched pathways closely associated with tumors. GSVA scoring was first performed on all tumors, and the correlation between PXDN expression and the pathway score in each tumor was calculated. This was then visualized using the R package “pheatmap.”

Red and blue represented positive and negative correlation, respectively; the darker the color, the stronger the correlation. The number of “*” corresponded to the magnitude of the p value.

### 2.7 Correlation analysis between PXDN and tumor microenvironment

Based on ssGSEA analysis results, we used the R package “Estimation of STromal and Immune cells in MAlignant Tumor tissues using Expression data (ESTIMATE)” to score the matrix gene sets and immune gene sets of all tumors. The sum of the two scores was used as the ESTIMATE score to finally obtain the relevant tumor purity.

Subsequently, we continued to explore the possible tumor microenvironment pathways involving PXDN, and used the research results of Dr. Zeng and his colleagues (including those related to immune-related pathways, epithelial-mesenchymal transformation, and DNA loss and repair pathways) (14), and obtained a correlation between PXDN gene expression and the score of different gene sets in turn; these results were then visualized using the R package “ggplot2.”

In the above visualization results, Red and blue represented positive and negative correlation, respectively; the darker the color, the stronger the correlation. The number of “*” corresponded to the magnitude of the p value.

### 2.8 Correlation analysis between PXDN and immune infiltration

We also investigated the relationship between PXDN and fibroblast or related immune cell infiltrates; raw related infiltrate data were obtained from the TIMER2 online database (version 2, http://timer.cistrome.org/) and the ImmuCellAI (http://bioinfo.life.hust.edu.cn/ImmuCellAI#!/) database. Three different algorithms, “EPIC, MCPCOUNTER, XCELL,” were used to analyze the correlation between PXDN and associated fibroblast infiltration, and six algorithms (TIMER, EPIC, MCPCOUNTER, CIBORSOFT, CIBORSOFT-ABS, and QUAN) were used to analyze the correlation between PXDN and associated immune cells infiltration; the results were visualized using the R package “ggplot2”.

Finally, we explored the relevance of PXDN to immune-related genes, including immune-related suppressor-related genes and immune-related activator genes in tumors, and chemokine-related, chemokine receptor-related, and MHC-related genes. The R package “ggplot2” was used to visualize the results.

In the above visualization results, Red and blue represented positive and negative correlation, respectively; the darker the color, the stronger the correlation. The number of “*” corresponded to the magnitude of the p value.

### 2.9 Analysis of PXDN and tumor mutational burden and microsatellite instability

TMB is defined as the total number of somatic gene coding, base insertion, substitution, or deletion errors detected per million bases (15), and it is an important biological indicator of the degree of mutation in tumors; a higher TMB is usually related to a better tumor immunotherapy outcome (16). MSI is caused by a defect in the DNA mismatch repair function within tumor tissue, and MSI-H tumors often have better treatment outcomes (17). The TMB-related data were obtained from relevant gene mutation data in the UCSC Xena database, and the MSI-related data were obtained from the research of Bonneville et al. (18). Finally, the results were visualized as a radar graph using the R package “fmsb”, where * represented the p value.

### 2.10 Analysis of PXDN drug resistance

Finally, we used relevant data from the GDSC2 (https://www.cancerrxgene.org/) database to explore the correlation between PXDN and drug IC50, and 198 drugs were included. An RNAseq data analysis was performed using the cell line expression profile data from GDSC. We plotted the difference in the IC50 for each drug in the high and low PXDN gene expression groups, separately, where * represented the p-value.

## 3 Results

### 3.1 Results of PXDN expression analysis

**Figure 1:**
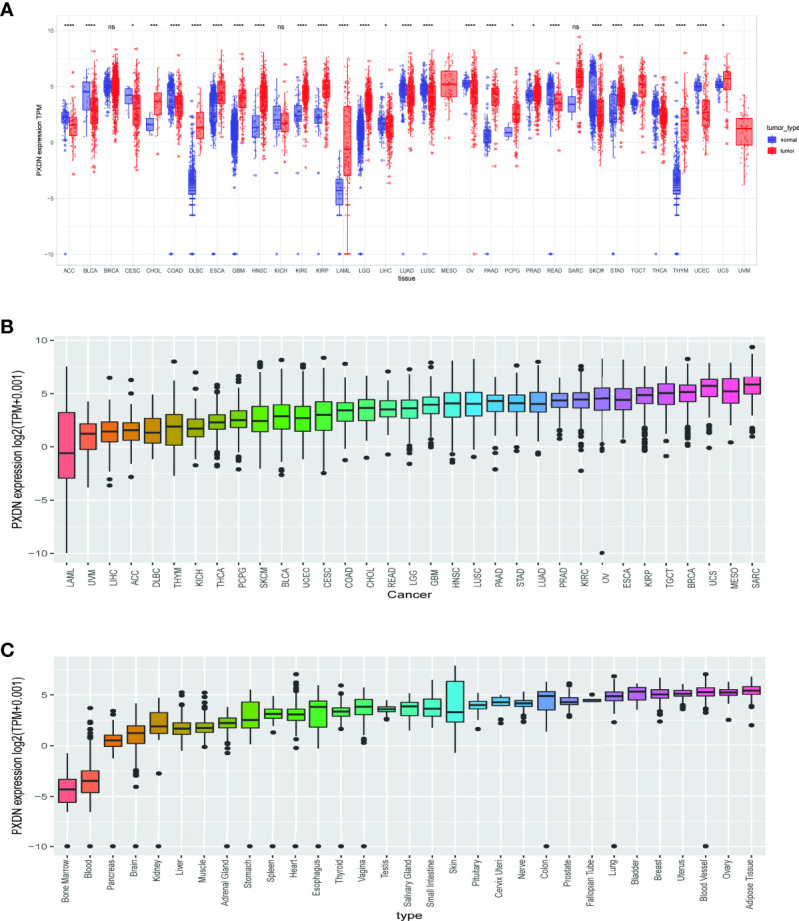
Expression of PXDN gene in different tumours and normal tissues. The results are based on data collated from the TCGA and GTEx databases. **(A)** Analysis of differential expression of PXDN in tumor tissues and normal tissues. *p < 0.05, ***p < 0.001, ****p < 0.0001. **(B)** The differences of the PXDN expression increased from left to right in each of the 33 tumors. **(C)** The differences of the PXDN expression increased from left to right in each of the 31 normal tissues. ns, no significance.

To clarify the differential expression of PXDN in tumor tissues versus normal tissues in different tumors, we combined the PXDN expression profile data of all samples obtained from the TCGA database with those of GTEx data to explore the difference of PXDN mRNA expression. As shown in **Figure 1A**, with the exception of MESO and Uveal Melanoma (UVM), for which there were no corresponding normal tissue controls, PXDN was not significantly differentially expressed in three tumors, Breast invasive carcinoma (BRCA), Liver hepatocellular carcinoma (LIHC), and SARC, but it was differentially expressed in the remaining 31 tumors, and all of the differences were statistically significant. We then ranked the PXDN expressions of each tumor and normal tissues in descending order; as shown in **Figure 1B**, the lowest and highest PXDN expressions occurred in Acute Myeloid Leukemia (LAML) and SARC, respectively. As shown in **Figure 1C**, the PXDN expression was significantly lower in normal human bone marrow and blood than in other tissues, and the highest expression was found in the normal adipose tissue.

**Figure 2:**
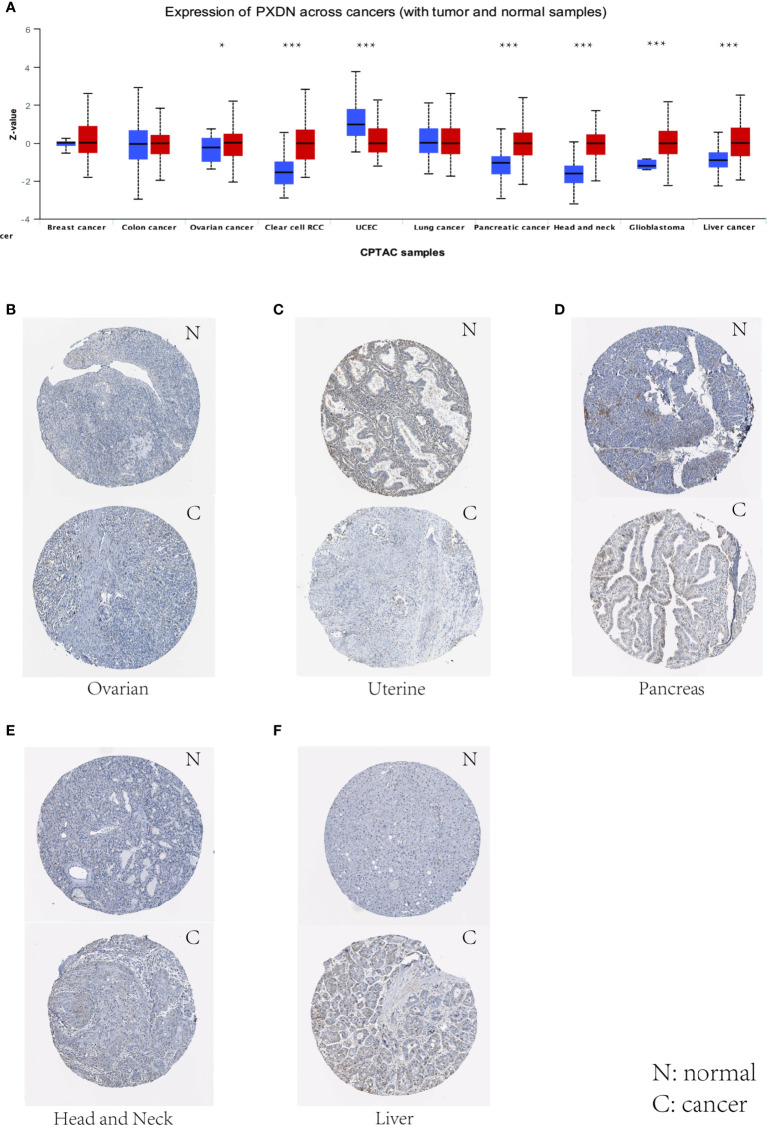
Levels of PXDN in different tumours. Results based on CTPAC and HPA database. **(A)** PXDN is differentially expressed in breast cancer, ovarian cancer, colon cancer, clear cell renal cell carcinoma (RCC), UCEC, lung cancer, pancreatic cancer, head and neck carcinoma, glioblastoma and liver cancer in tumour tissues and normal tissues in CTPAC database. **(B–F)** The IHC of PXDN in ovarian cancer, pancreatic cancer, head and neck carcinoma and liver cancer was much higher than that in normal tissues. *p < 0.05, ***p < 0.001.

We also assessed the expression levels of PXDN at the protein level. We analyzed the protein expression differences of PXDN in 10 tumors using the CPTAC database and compared the results with the IHC staining results from the HPA database. As shown in **Figure 2A**, there were significant differences in the PXDN protein expressions of seven tumors, including those of ovarian cancer, RCC, UCEC, pancreatic cancer, head and neck carcinoma, glioblastoma, and liver cancer. With the exception of UCEC, in which the PXDN protein expression level of normal tissue was high, PXDN protein was highly expressed in the other six tumor types. The results of subsequent immunohistochemical staining were approximately similar to the above results, as shown in **Figures 2B**–**F**. With the exception of the lower significant difference in the relevant staining of uterine tumor samples and normal samples, the level of immunohistochemical staining in the rest of the tumor tissues was medium, while that in the corresponding normal tissues was low.

### 3.2 Results of prognostic analysis

**Figure 3:**
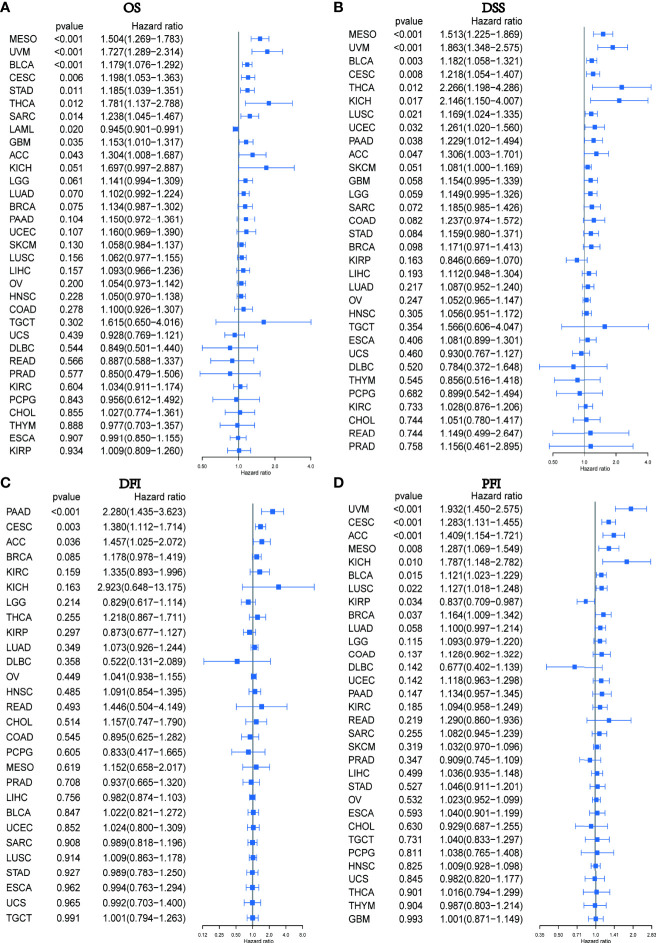
Relationship between PXDN expression and OS, DSS, DFI and PFI. **(A)** Expression of PXDN is associated with OS in 33 tumour types. **(B)** Expression of PXDN is associated with DSS in 32 tumour types. **(C)** Expression of PXDN is associated with DFI in 32 tumour types. **(D)** Expression of PXDN is associated with PFI in 32 tumour types.

We subsequently conducted a univariate COX analysis based on the PXDN gene expression levels of the samples in combination with their clinical data, and assessed the correlation between PXDN and prognosis using four indicators in turn (OS (including 33 tumors), DSS (including 32 tumors, lacking LAML), DFI (including 32 tumors, lacking LAML), and PFI [including 32 tumors, lacking LAML)] and then ranked the correlation scores from lowest to highest according to the p-value. As shown in **Figure 3A**, there was a close correlation between OS and the PXDN expression in 10 tumors. PXDN acted a protective factor in LAML (p < 0.001) but was a risk factor in the other tumors, including MESO (p < 0.001), Bladder Urothelial Carcinoma(BLCA) (p < 0.001), Cervical squamous cell carcinoma and endocervical adenocarcinoma (CESC, p < 0.006), Stomach adenocarcinoma (STAD, p=0.011), Thyroid carcinoma (THCA, p=0.012), SARC (p=0.014), Glioblastoma multiforme (GBM, p=0.035), and Adrenocortical carcinoma (ACC, p=0.043). As shown in **Figure 3B**, DSS was correlated with 10 of these tumors, namely, MESO (p < 0.001), UVM (p < 0.001), BLCA (< 0.003), CESC (p=0.008), THCA (p=0.012), Kidney Chromophobe (KICH, p=0.017), Lung squamous cell carcinoma (LUSC, p=0.021), UCEC (p=0.032), Pancreatic adenocarcinoma (PAAD, p=0.038), ACC (p=0.047), and was a risk factor for all of them. However, DFI was only significant and served as a risk factor in three types of tumors: PAAD (p < 0.001), CESC (p = 0.003), ACC (p = 0.036), as shown in **Figure 3C**. Finally, as shown in **Figure 3D**, the PXDN expression was significantly correlated with PFI in 9 tumors. With the exception of its expression in Kidney renal papillary cell carcinoma (KIRP) as a protective factor, it was a risk factor in UVM (p < 0.001), CESC (p < 0.001), ACC (p < 0.001), MESO (p < 0.008), KICH (p=0.010), BLCA (p=0.015), LUSC (p=0.022), and BRCA (p=0.037). The specific Kaplan-Meier survival curve is shown in **Figure S1** in detail.

**Figure 4:**
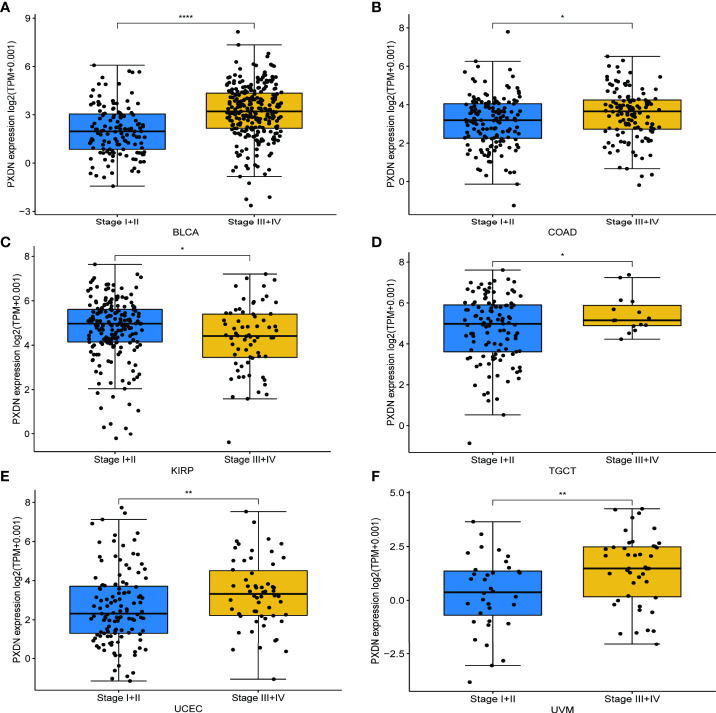
Relationship between PXDN expression and different pathological stages. **(A–F)** Relationship between PXDN levels and different pathological stages of BLCA, COAD, KIRP, TGCT, UCEC, UVM. *p<0.05, **p<0.01, ****p<0.0001.

We also discussed the effect of PXDN expression on tumor stage. As shown in **Figures 4A–F**, with the exception of KIRP, there was a higher PXDN expression in BLCA, Colon adenocarcinoma (COAD), Testicular Germ Cell Tumors (TGCT), UCEC, and UVM that often implied a higher staging, and the p-values were statistically significant. The correlations between PXDN and tumor stages in the remaining tumors are detailed in **Figure S2**.

### 3.3 Results of PXDN methylation analysis

**Figure 5:**
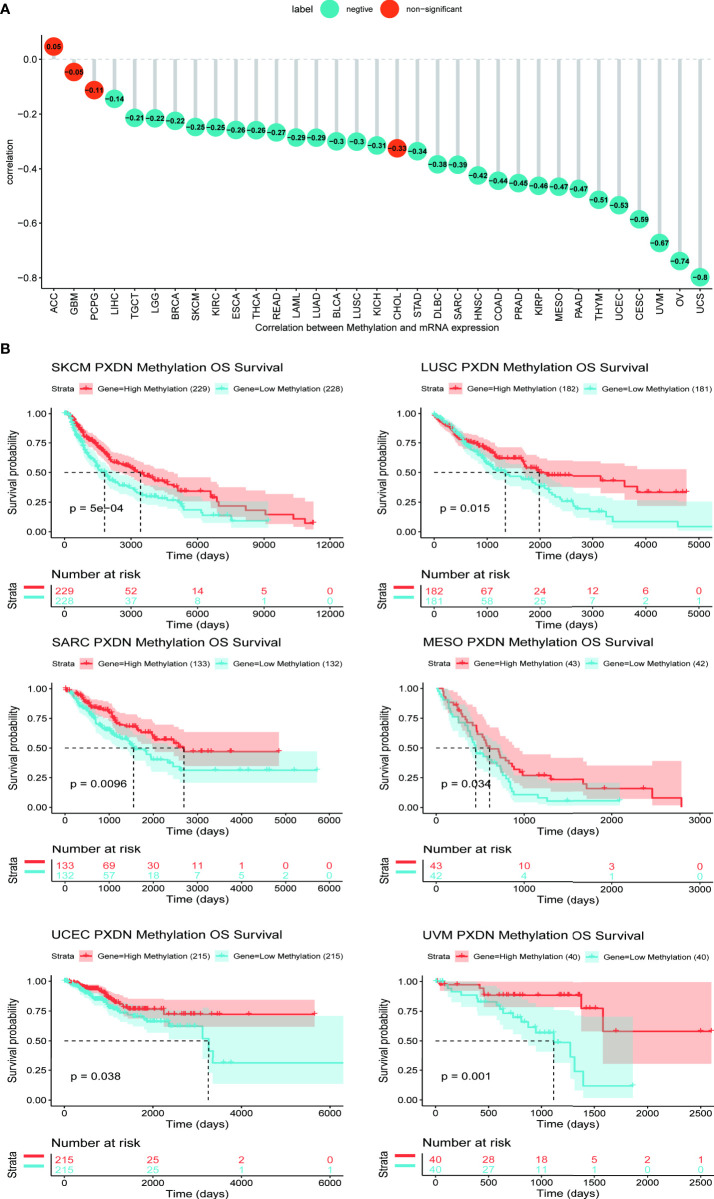
Relationship between PXDN expression and promoter methylation in different tumours. The results are based on data collated from cBioPortal database. **(A)** Relationship between PXDN expression and promoter methylation in 33 tumour types. **(B)** Kaplan-Meier survival analysis of the relationship between PXDN promoter methylation level and OS in SKCM, LUSC, SARC, MESO, UCEC, UVM.

Studies have found that high methylation levels often lead to the downregulation of mRNA. We therefore analyzed the correlation between PXDN expression and its methylation level using data from the cBioPortal database. As shown in **Figure 5A**, with the exception of four tumors (ACC, GBM, Pheochromocytoma and Paraganglioma (PCPG), and Cholangiocarcinoma (CHOL)), there was an evident negative correlation between the PXDN expression and the methylation level in all other tumors. We subsequently analyzed the effect of the PXDN methylation level on tumor prognosis, and obtained Kaplan-Meier survival curves for different methylation levels in different tumors. As shown in **Figure 5B**, low methylation levels in Skin Cutaneous Melanoma (SKCM), LUSC, SARC, MESO, UCEC, and UVM often implied a worse prognosis, and the p-value was statistically significant. The Kaplan-Meier survival curves of the methylation levels of the remaining tumors are detailed in **Figure S3**.

### 3.4 Results of PXDN mutation analysis

**Figure 6:**
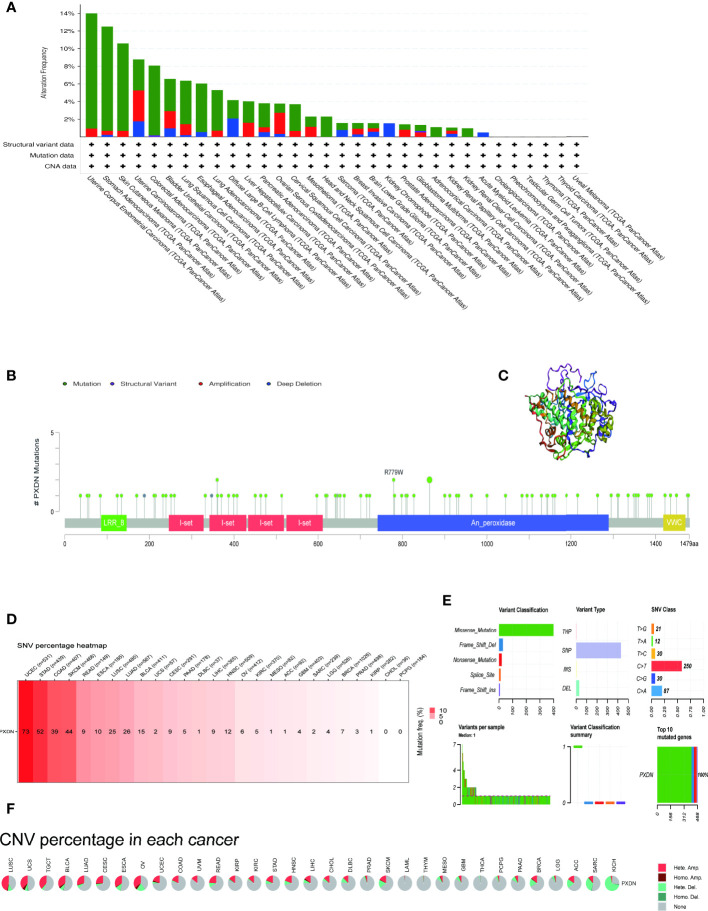
Mutation characteristics of PXDN gene in different tumours. The results are based on data collated from cBioPortal and GSCAlite database. **(A)** Types of mutations of PXDN in different tumours. **(B)** Frequency of PXDN mutation in different tumours. **(C)** Display the 3D structure of PXDN. **(D)** Frequency of SNV in 27 cancer types. **(E,A)** Top left (variant classification): count of each type of deleterious mutation (missense mutation, nonsense mutation, frame shift ins, splice site, frame shift del, in frame del, in frame ins) of PXDN in different tumors. Top middle (variant type): count of the SNP and DEL of PXDN in different tumors. Top right (SNV class): count of each SNV class of PXDN in different tumors. Down left (variants per sample): count of variants in each sample. A bar indicates a sample, and the color of the bar corresponds to the color of the variant classification. Down middle (variant classification summary): the distribution of the count of each variant classification in the sample set of different tumors. The color of the box corresponds to the color of the variant classification. Down right (top 10 mutated genes): count and percentage of variants in the top 10 mutated genes. **(F)** CNV of PXDN in the 33 cancer types.

We continued to utilize the cBioPortal database to observe PXDN gene mutations in different TCGA tumors. As shown in **Figure 6A**, where green color represents mutation, blue represents deep deletion, and red represents amplification, the mutation rate of PXDN reached its highest level of 14% in UCEC, whereas there were no mutations in CHOL, PCPG, TGCT, THCA, Thymoma (THYM), or UVM. All the genetic loci and the number of PXDN cases are shown in **Figures 6B**, **C** shows a 3D map of PXDN.

We subsequently explored the relationship between PXDN and SNV and CNV. **Figure 6D** shows an overall picture of SNV in a tumor. Among the 531 samples in UCEC, 73 samples showed SNV phenomenon, but none of the CHOL and PCPG samples contained SNV. Moreover, the missense mutations and base C mutations rates of most samples were greater than the remaining mutation types and base mutations combined, as shown in **Figure 6E**. **Figure 6F** shows that the CNV type varied in different tumors. For example, in LIHC, nearly 50% of the CNV types were “Hete.Amp.”, whereas in KICH, more than 50% of the CNV types were “Hete.Del”. In contrast, CNV was largely absent in tumors such as LAML and THYM.

### 3.5 Results of GSEA and GSVA analyses

**Figure 7:**
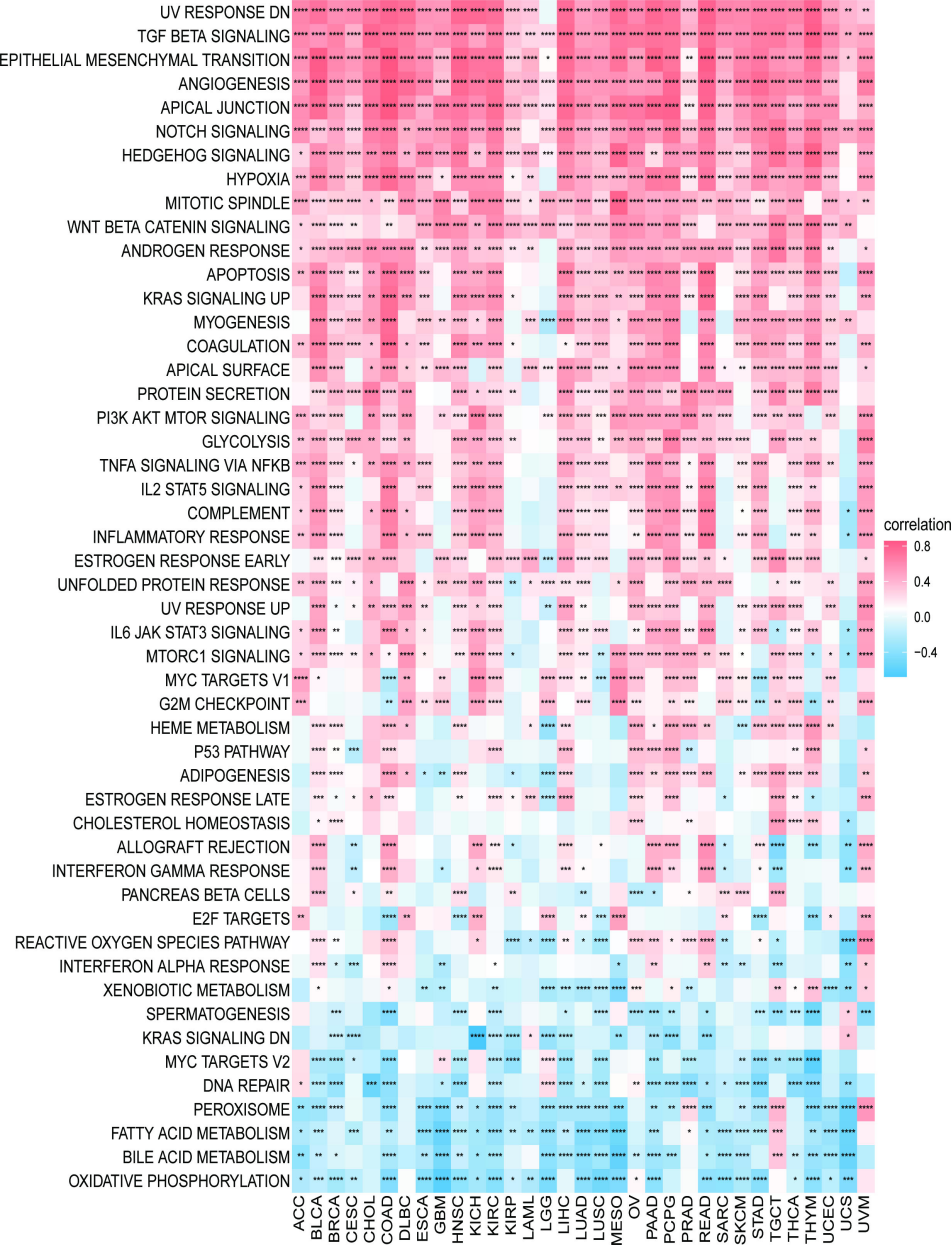
GSVA results. Results of HALLMARK enrichment analysis of PXDN in different tumors. Red and blue represent positive and negative correlations, respectively, while white represents p > 0.05. *p < 0.05, **p < 0.01, ***p < 0.001, ****p < 0.0001.

We first explored the possible enrichment pathways of PXDN in 33 tumors using the GSEA approach, and the results of GO gene-set based analysis are shown in **Figure S4**. To further explore the underlying molecular mechanisms and the possible role of PXDN in tumors, we then conducted an analysis using the GSVA approach. The results based on the HALLMAKER gene set are shown in **Figure 7**, where it is evident that PXDN is correlated with almost all tumors, especially in the “UV RESPONSE DN”, “TGF BETA SIGNALING”, “ANGIOGENESIS”, “APICAL JUNCTION”, “NOTCH SIGNALING”, “HEDGEHOG SIGNALING” and “HYPOXIA” and other related enrichment pathways.

### 3.6 Results of correlation analysis between PXDN and tumor microenvironment

**Figure 8:**
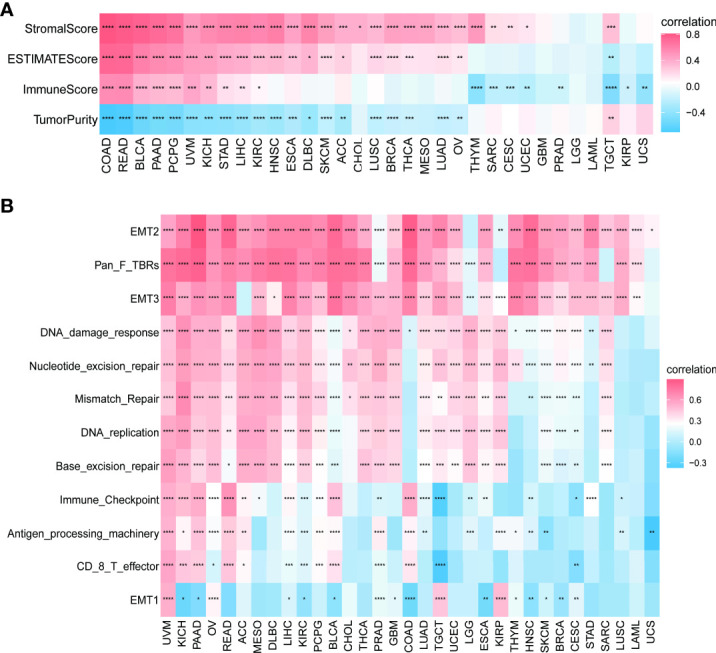
Relationship between PXDN expression and the tumor microenvironment. **(A)** Correlation between PXDN expression and stromal and immune scores. **(B)** Correlation between PXDN expression and tumor microenvironment-related pathways. Red and blue represent positive and negative correlations, respectively, while white represents p > 0.05. *p < 0.05, **p < 0.01, ***p < 0.001, ****p < 0.0001.

We continued to explore the relationship between PXDN and the tumor microenvironment. As shown in **Figure 8A**, a positive correlation was determined between PXDN and the stromal microenvironment of most tumors, while the results of correlations between PXDN and the immune microenvironment differed or were completely opposite between different tumors. We then explored the possible PXDN action mechanisms within the tumor microenvironment. As shown in **Figure 8B**, PXDN was positively correlated with pathways such as epithelial mesenchymal transition in almost all tumors.

### 3.7 Results of correlation analysis between PXDN and immune infiltration

**Figure 9:**
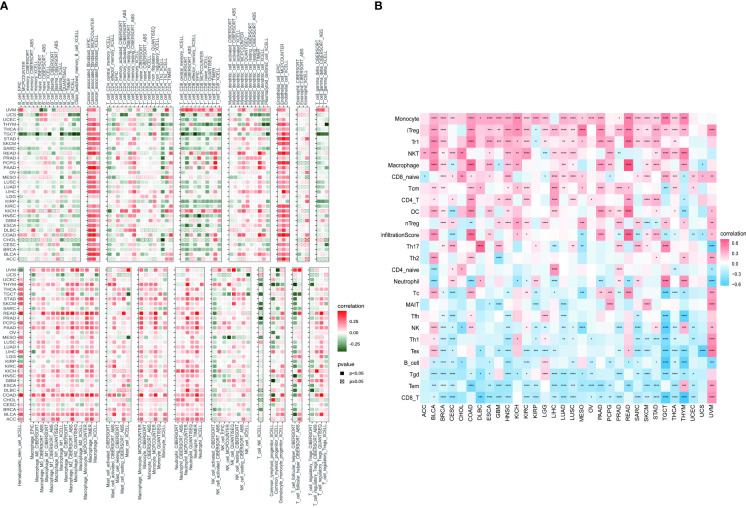
Relationship between PXDN expression and tumor immune cell infiltration. **(A)** Relationship between PXDN expression and tumor immune cell infiltration based on the TIMER2 database. **(B)** Relationship between PXDN expression and tumor immune cell infiltration based on the ImmuneCellAI database. Red and blue or green represents positive and negative correlations, respectively, while white represents p > 0.05. *p < 0.05, **p < 0.01, ***p < 0.001, ****p < 0.0001.

Based on the findings of the role of PXDN in the tumor microenvironment, we conducted an in depth investigation into the relationship between PXDN and specific immune cell infiltration. According to data from the TIMER2 database, the increased PXDN expression was accompanied by a large infiltration of macrophages and neutrophils in almost all tumors, but the opposite was true for CD8+ T cells (**Figure 9A**). Subsequent results also indicated that fibroblast infiltration was positively correlated with PXDN expression in almost all tumors. We then continued to use the data from the ImmuCellAI database to further refine the relevant analysis. As shown in **Figure 9B**, the analysis of CD8+ T cell infiltration provided similar results when using the data from both databases. Although this result indicated that a high PXDN expression in tumor tissues was accompanied by the infiltration of large numbers of immune cells, the specific types of immune cells involved required specific tumor analysis.

**Figure 10:**
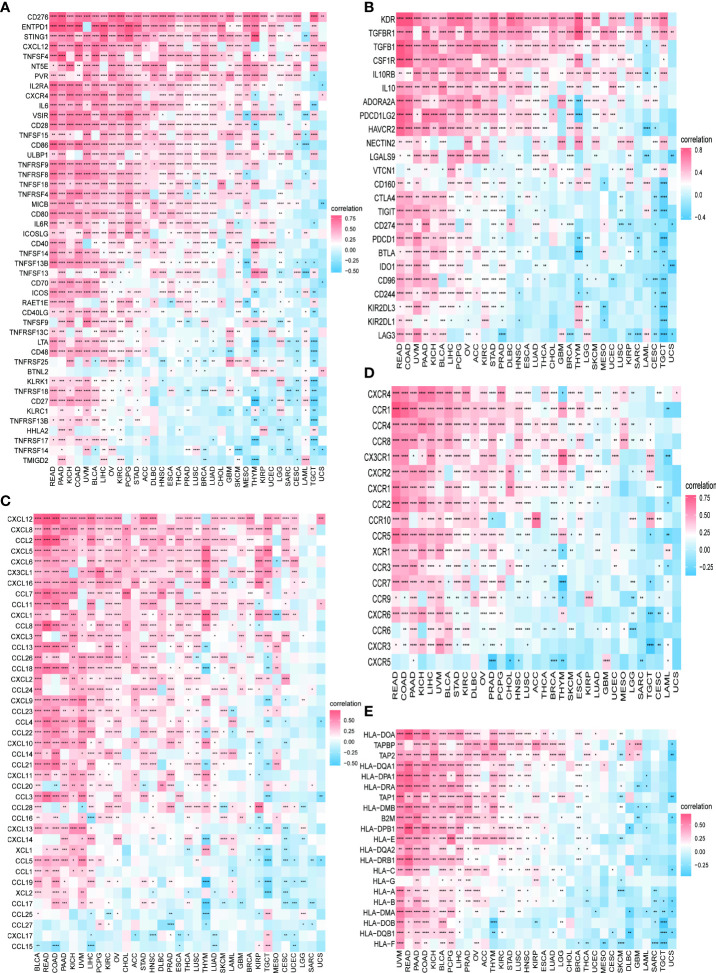
Relationship between PXDN expression and immune-associated genes. **(A)** Relationship between PXDN expression and the immuno-associated activating genes. **(B)** Relationship between PXDN expression and the immuno-associated suppressive genes. **(C)** Relationship between PXDN expression and the genes-related chemokine. **(D)** Relationship between PXDN expression and the genes-related chemokine receptor. **(E)** Relationship between PXDN expression and the MHC genes. Red and blue represent positive and negative correlations, respectively, while white represents p > 0.05. *p < 0.05, **p < 0.01, ***p < 0.001, ****p < 0.0001.

We further continued to explore the relationship between PXDN and immune-related genes. As shown in **Figures 10A, B**, with the exception of a few immune activation-related genes and immunosuppression-related genes, most of the other genes were positively correlated with PXDN in the pan-cancer analysis. However, chemokine-related genes, chemokine receptor-related genes, and MHC-related genes, (as shown in **Figures 10C–E**) require specific gene analyses, and the results vary for each tumor.

### 3.8 Results of correlation between PXDN and TMB or MSI

**Figure 11:**
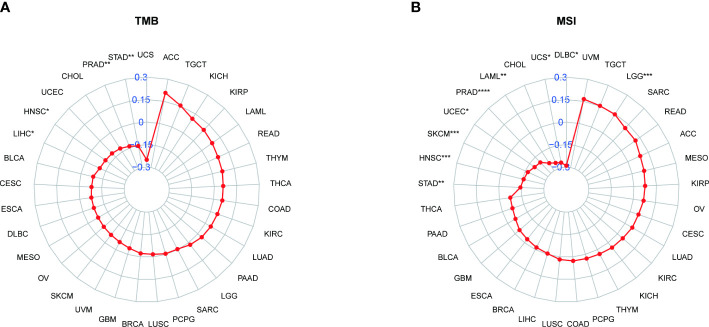
Relationship between PXDN expression and TMB and MSI. **(A)** Radar plot of the correlation between PXDN expression and TMB in different tumors. **(B)** Radar plot of the correlation between PXDN expression and MSI in different tumors. *p < 0.05, **p < 0.01, ***p < 0.001, ****p < 0.0001.

We also analyzed the correlation between PXDN and TMB or MSI. As shown by **Figure 11A**, the expression of PXDN was negatively correlated with TMB in LIHC, Head and Neck squamous cell carcinoma (HNSC), Prostate adenocarcinoma (PRAD), and STAD, and the p-values were statistically significant. For MSI, there was also a negative correlation between the expression of PXDN and tumors, including STAD, HNSC, SKCM, UCEC, PRAD, LAML, Uterine Carcinosarcoma (UCS), Lymphoid Neoplasm Diffuse Large B-cell Lymphoma (DLBC) and others, while the opposite was true in Brain Lower Grade Glioma, as shown in **Figure 11B**.

### 3.9 Results of PXDN drug resistance analysis

**Figure 12:**
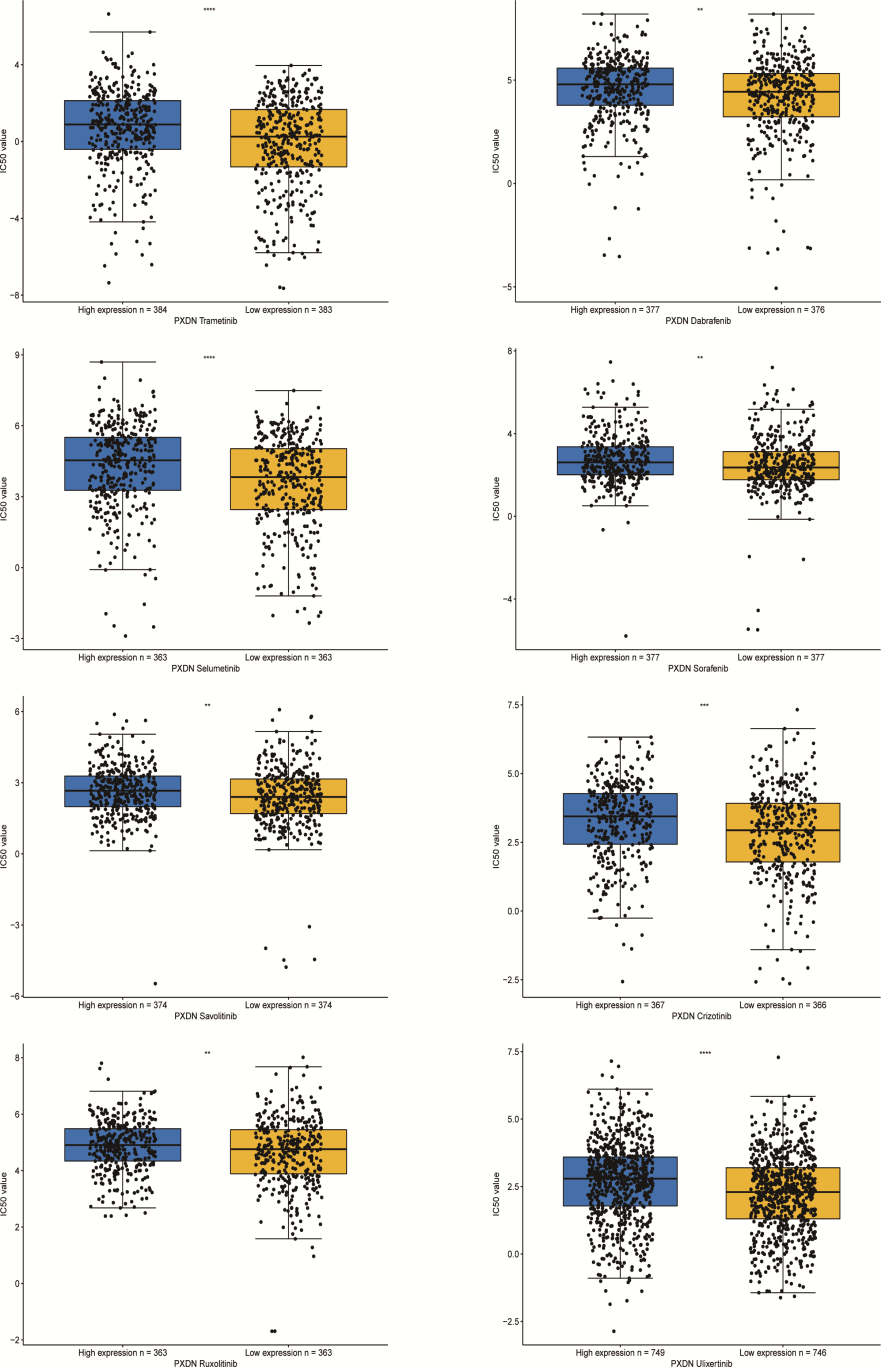
IC50s for different therapeutic drugs in the PXDN expression subgroups. **p < 0.01, ***p < 0.001, ****p < 0.0001.

Finally we explored the sensitivity of PXDN to clinically targeted therapeutic agents using data from the GDSC2 database. Some common targeted therapeutic agents such as sorafenib were selected, and they were grouped according to PXDN expression levels. As shown in **Figure 12**, we can see that the IC50 of the targeted drugs was significantly higher in the PXDN high expression group than in the PXDN low expression group.

## 4 Discussion

Several recent studies have determined an important link between PXDN and tumors, although it is also known to participate in normal physiological activities within the body. However, studies have not yet determined whether PXDN is clinically relevant or which specific mechanisms are employed in tumors. It is also unclear whether certain mechanisms exert the same or different effects in different tumors. The authors of this paper found no previous pan-cancer analysis of PXDN, and thus used data from several databases, including TCGA and GTEx, to conduct an in-depth analysis of PXDN and study its characteristics within tumors.

According to the results of our analysis of the expression of PXDN in tumor tissues and corresponding normal tissues, significant differences were found in 31 tumor types. However, its expression differed between high and low depending on the specific tumor type. A subsequent analysis of its expression at the protein level yielded essentially the same results, and we can thus broadly conclude that PXDN may be acting at both the gene and protein level. We also investigated the relationship between PXDN and prognosis. According to our results, PXDN tends to promote the development of prostate and bladder cancer, and this agrees with previous findings (19, 20). Our results also show that PXDN tends to play a pro-cancer role in tumors. Further studies of its clinically relevant traits subsequently determined that a high PXDN expression is often accompanied by a higher stage in prostate cancer, and this could be one of the reasons associated with a poor prostate cancer prognosis. For the remaining tumor types, it is reasonable to assume that they have the same clinical value.

However, studying PXDN from the gene expression level and prognosis aspect was insufficient, and we thus explored the different methylation levels of PXDN in tumors. DNA methylation is an important epigenetic mechanism that controls cell proliferation, apoptosis, differentiation, the cell cycle, and transformation in eukaryotes, and many cancers are closely related to promoter methylation (21). We were thus pleasantly surprised to find that PXDN was negatively correlated with its methylation levels in all tumors, except in those with no statistical significance. This study is also the first to analyze the effect of the PXDN methylation level on prognosis and report that hypermethylation of PXDN tends to represent longer patient survival times. As some studies have found that the PXDN methylation level is significant in pancreatic cancer (22) and colorectal tumors (23), we thus believe that the PXDN methylation level can be used as an independent predictor of tumor.

We also studied the overall mutation of PXDN and the specific conditions of SNV and CNV. Our results showed that PXDN has high mutation and SNV levels in UCEC. However, we previously determined that PXDN is often expressed at a low level in UCEC tumors; therefore, its low expression level but high mutation rate requires further study. There is a currently a need for studies investigating whether various CNVs (including this gene) have the same effect on tumor development in different tumors, or whether the effect is completely different, and increased data analysis and the subsequent sharing of data and results are required to clarify effects on tumor susceptibility.

We further analyzed the possible relevant molecular mechanisms involved in the action of PXDN in tumors. According to our results, PXDN plays a role through certain classical tumor pathways in most tumors, such as the “TNF BETA SIGNALING” or “PI3K AKT MTOR SINALING” pathways. As previously mentioned, studies have found that PXDN may play a role in ovarian cancer by regulating the PI3K/Akt pathway (11). We were also pleasantly surprised to find a close link between PXDN and the immune response and other pathways.

We further investigated the relationship between PXDN and the tumor microenvironment, including the stromal and immune microenvironments. Studies have shown that PXDN itself is involved in the formation of extracellular mesenchyme (7), and our results also showed an increase in stromal microenvironment scores in tumors with an increased PXDN expression. PXDN is also involved in related pathways within the tumor microenvironment, including epithelial-mesenchymal transformation. However, the PXDN scores of the immune microenvironment were potentially opposite in different tumors, and this result was thought-provoking. We thus used data from two databases to explore the correlation between specific immune cells infiltrating different tumors and the expression profile of PXDN. Our results showed that the PXDN expression was positively correlated with tumor-associated fibroblast infiltration in almost all tumors, and this result was also consistent with that of previous findings. The PXDN expression was also positively correlated with macrophage and neutrophil infiltration, but negatively correlated with CD8+ T cells, which are tumor-killing cells. This may imply that immunotherapy will not be favorable when PXDN is highly expressed, and it may be one of the mechanisms associated with the pro-cancer role of PXDN in most tumors.

To further elaborate the significance of PXDN in immunotherapy, we also explored the correlation between PXDN and tumor TMB and MSI. Studies have found that a high TMB often implies increased survival after immunotherapy (24), and our statistically significant results also found a negative correlation between PXDN and TMB in tumors. For MSI, there is ample evidence that patients with MSI-H in colorectal tumors benefit from immunotherapy (25), and our results also revealed a negative correlation between PXDN and MSI in most tumors, including gastric cancer. This may also be one of the potential mechanisms found in previous studies by which PXDN can act as an independent risk factor in gastric cancer (26).

We finally verified the clinical value of PXDN by conducting a drug resistance analysis. Of almost all targeted therapeutics, the IC50 level was significantly higher in the PXDN high expression group than in the low expression group. This may imply that patients with a high PXDN expression are more likely to develop drug resistance when receiving treatment and are less likely to benefit from such treatment.

## 5 Conclusion

Peroxidasin (PXDN) is a newly discovered heme-containing peroxidase that regulates redox reactions in the body. Recent studies have revealed that PXDN plays a role in tumors, and as such it is receiving increasing attention. We aimed to conduct a pan-cancer analysis to explore the correlation between many aspects of PXDN and tumors. Although certain limitations exist, such as the limited amount of relevant studies consulted, insufficient persuasive power, and a lack of relevant experimental validation, our results show that PXDN potentially has certain high effects in tumors, including its effects on the tumor microenvironment and immune cell infiltration. These findings will assist in further elucidating the role of PXDN in tumorigenesis and progression and can be used as a new reference for its potential application in immunotherapy. And follow-up studies can be combined with immune checkpoint inhibitors and clinical studies, to further find the value of PXDN.

## Data availability statement

The original contributions presented in the study are included in the article/**Supplementary Material**. Further inquiries can be directed to the corresponding authors.

## Author contributions

XHZ and YJS designed the study. XHZ and ZZ performed the majority of the experiments and analyzed the data. XPZ and ZSH analyzed and checked the data. QS and CX wrote the manuscript supervised by WLW, and YJS. All authors contributed to the article and approved the submitted version.

## Funding

This work was supported by The National Natural Science Foundation of China (No. 82072203) and Natural Science Foundation of Zhejiang Province (No. LQ19H160025).

## Conflict of Interest

The authors declare that the research was conducted in the absence of any commercial or financial relationships that could be construed as a potential conflict of interest.

## Publisher’s note

All claims expressed in this article are solely those of the authors and do not necessarily represent those of their affiliated organizations, or those of the publisher, the editors and the reviewers. Any product that may be evaluated in this article, or claim that may be made by its manufacturer, is not guaranteed or endorsed by the publisher.

## Supplementary material

Supplementary Figure 1 Kaplan-Meier survival analysis of the relationship between expression of PXDN and OS, DSS, DFI and PFI in specific tumours.

Supplementary Figure 2 Expression of PXDN is related to the different pathological stages of other tumours in the TCGA database.

Supplementary Figure 3 Kaplan-Meier survival analysis of the relationship between PXDN promoter methylation level and OS in other tumours..

Supplementary Figure 4 GSEA results, based on PXDN-binding and correlated gene GO enrichment analysis results.
