# Supplementary figures and images for: A systematic pan-cancer analysis of PXDN as a potential target for clinical diagnosis and treatment
Source: Front Oncol. 2022 Aug 2;12:952849. doi: 10.3389/fonc.2022.952849 (PMC9380648; doi:10.3389/fonc.2022.952849)

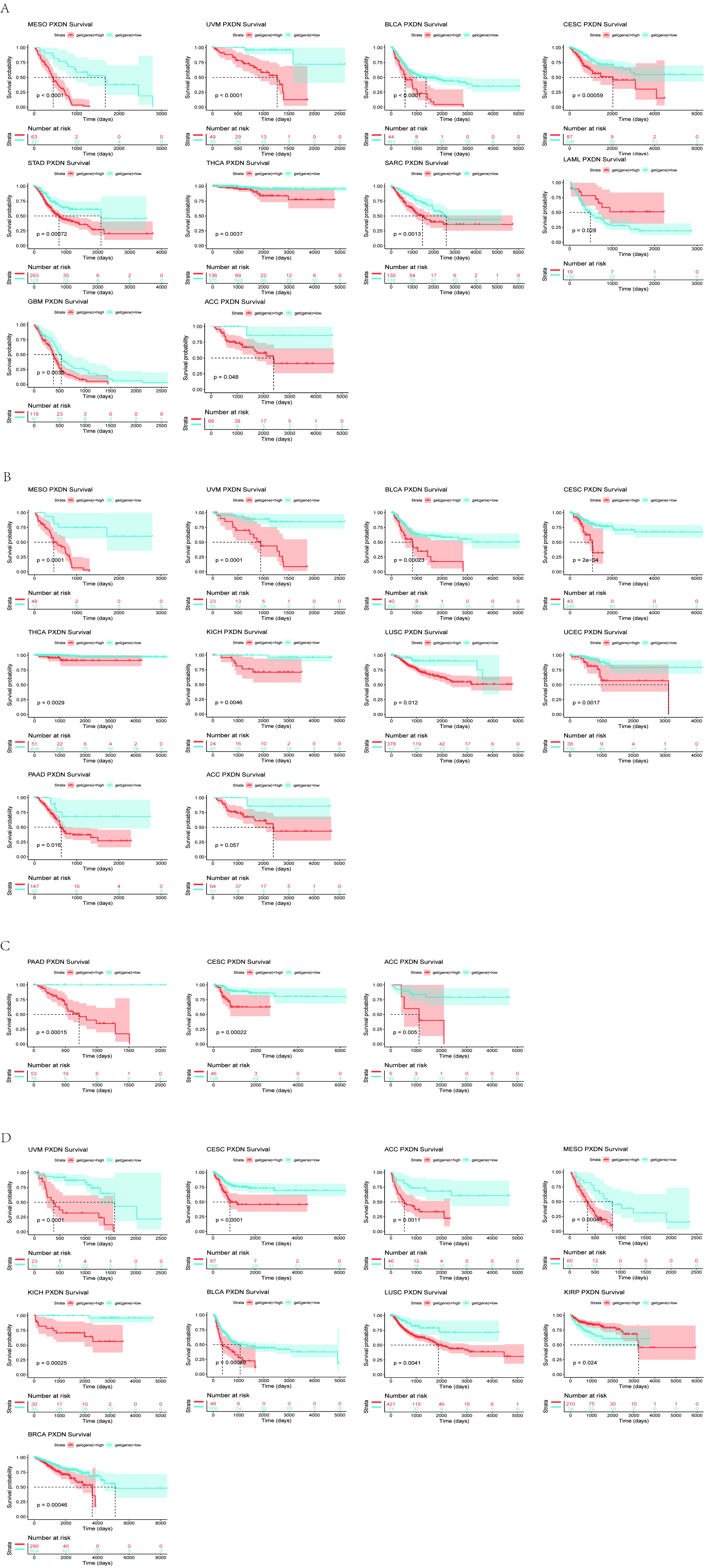

Supplement: Supplementary Figure 1 — Kaplan-Meier survival analysis of the relationship between expression of PXDN and OS, DSS, DFI and PFI in specific tumours. [file Image_1.jpeg]

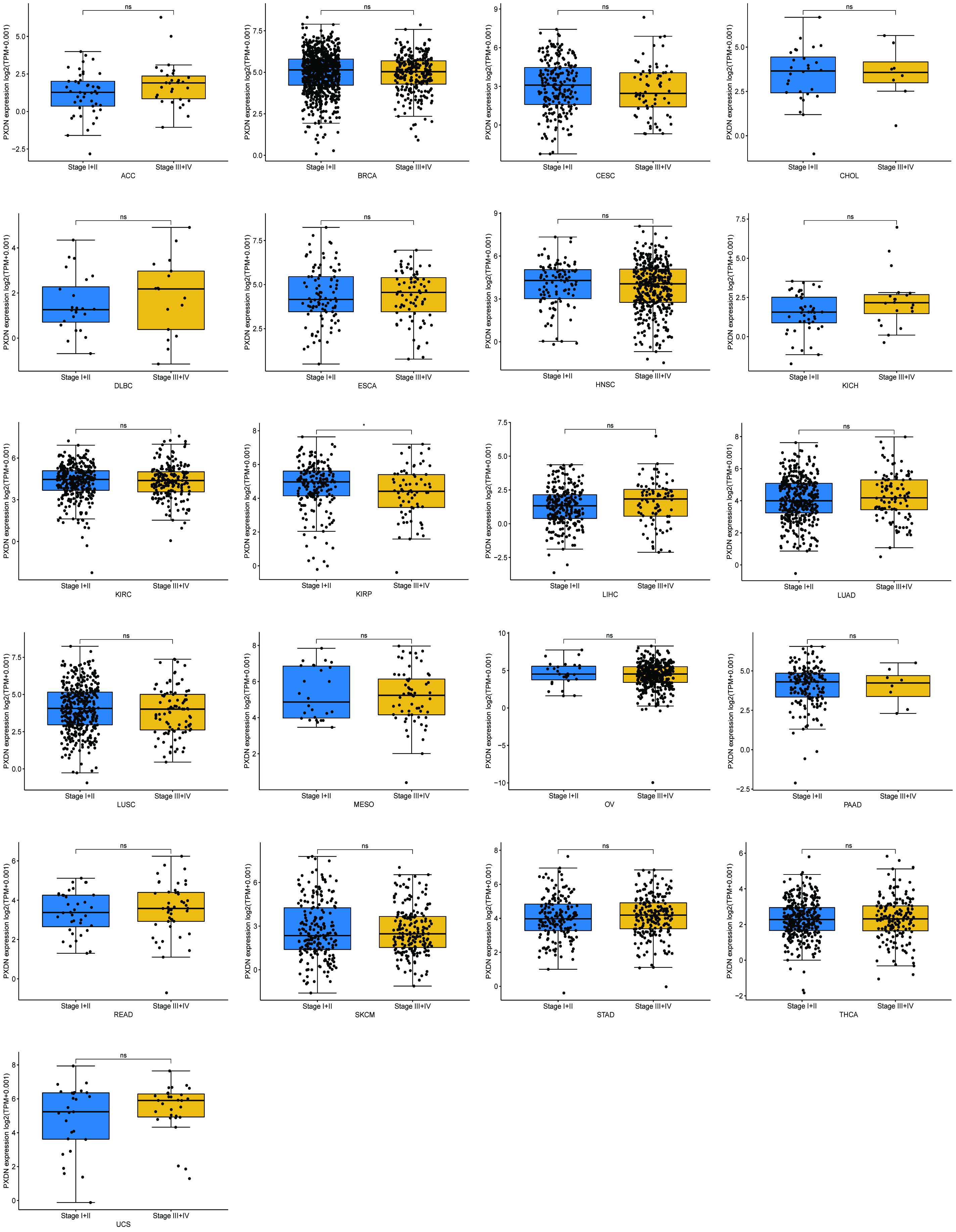

Supplement: Supplementary Figure 2 — Expression of PXDN is related to the different pathological stages of other tumours in the TCGA database. [file Image_2.jpeg]

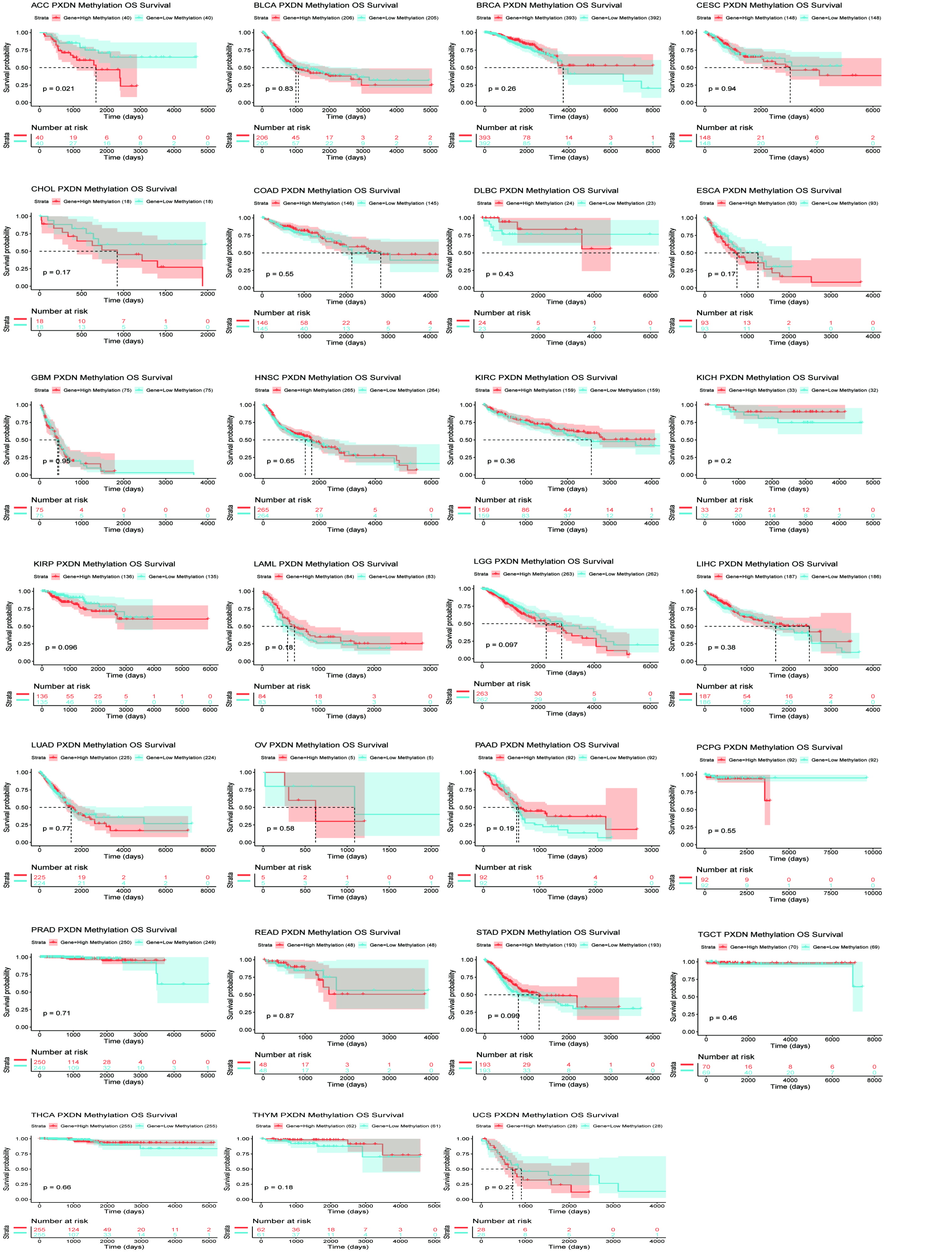

Supplement: Supplementary Figure 3 — Kaplan-Meier survival analysis of the relationship between PXDN promoter methylation level and OS in other tumours.. [file Image_3.jpeg]

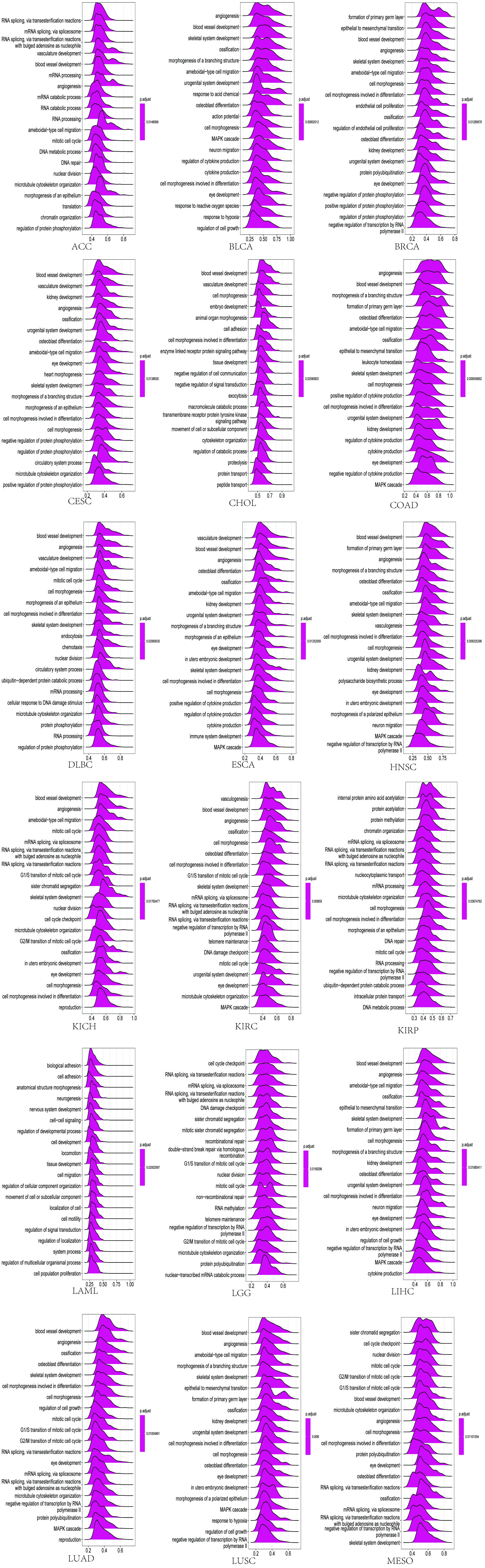

Supplement: Supplementary Figure 4 — GSEA results, based on PXDN-binding and correlated gene GO enrichment analysis results. [file Image_4.jpeg]

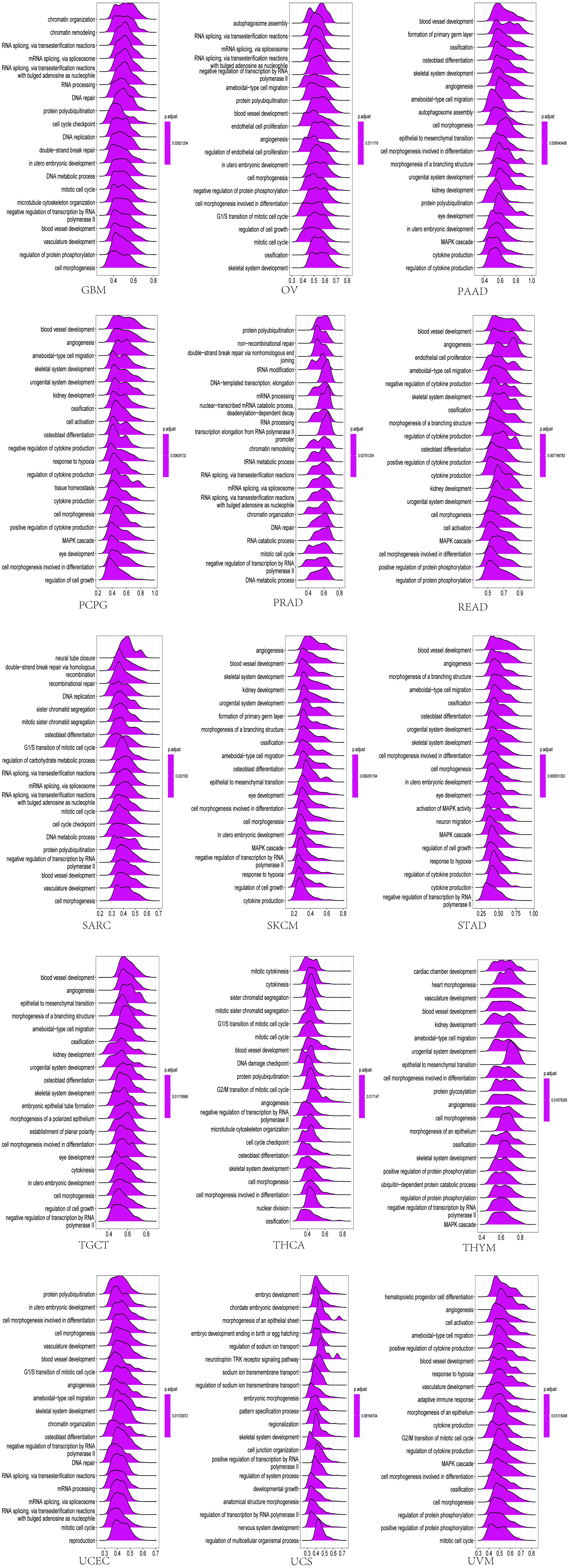

Supplement: Supplementary file 5 [file Image_5.jpeg]
